# XCMS-METLIN: data-driven metabolite, lipid, and chemical analysis

**DOI:** 10.1038/s44320-024-00063-4

**Published:** 2024-09-19

**Authors:** Martin Giera, Aries Aisporna, Winnie Uritboonthai, Linh Hoang, Rico J E Derks, Kara M Joseph, Erin S Baker, Gary Siuzdak

**Affiliations:** 1 Leiden University Medical Center, Center for Proteomics and Metabolomics, Albinusdreef 2, 2333ZA Leiden, Netherlands; 2 The Novo Nordisk Foundation Center for Stem Cell Medicine (reNEW), Leiden University Medical Center, Leiden, the Netherlands; 3 Scripps Center of Metabolomics and Mass Spectrometry, La Jolla, CA 92037 USA; 4 Department of Chemistry, The University of North Carolina at Chapel Hill, Chapel Hill, NC 27599 USA; 5 Department of Chemistry, Molecular & Computational Biology Scripps Research, La Jolla, CA 92037 USA

**Keywords:** Metabolism, Methods & Resources

## Abstract

In this Correspondence, G. Siuzdak and colleagues present XCMS-METLIN, an extensive resource for metabolomics, lipidomics, and chemical analysis.

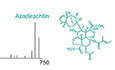

Tandem mass spectrometry (MS/MS)-based methods are essential in metabolomics, lipidomics, and chemical profiling, offering profound insights into biological systems and supporting advancements in fundamental biology, drug discovery, and personalized medicine (Heiles, 2021). Despite the realization that the metabolome plays an essential role in biology’s central dogma shaping cellular phenotype and function, the reliable identification of metabolites and chemical entities remains a significant bottleneck, limiting their integration into the expansive “omics” data landscape. To overcome this challenge, we have integrated the XCMS (eXtensible Computational Mass Spectrometry) data processing platform (Smith et al, 2006; Tautenhahn et al, 2012a) with the METLIN experimental MS/MS database (Smith et al, 2005; Tautenhahn et al, 2012a; Xue et al, 2020), which now hosts over 935,000 molecular standards. This integration marks a departure from platforms dependent on computationally derived molecular identifications, leveraging a data-driven approach that avoids speculative similarity networking based on in silico predictions. By enhancing the accuracy and scope of molecular identifications, the XCMS-METLIN platform not only strengthens data integrity but also paves the way for the incorporation of metabolomics into the realm of digital biology, thereby expanding its applicative potential across scientific disciplines.

Digital and chemical biology (Hudson et al, 2023; Gauthier et al, 2019) are pivotal in enhancing our comprehension of biological systems by integrating diverse “omics” datasets. Established fields such as genomics, transcriptomics, and proteomics have effectively utilized these data to unravel complex biological mechanisms and integrate them into the big data era. Metabolomics, which stands at the convergence of these disciplines, provides the most direct reflection of an organism’s phenotype. Despite its critical position, the field faces significant challenges in the comprehensive characterization of the metabolome (Bowen et al, 2010), with only a fraction of metabolites being successfully annotated. This limitation hinders the full potential of metabolomics to provide detailed insights into metabolic functions and processes. The impact of this challenge reaches far beyond biological research, affecting diverse sectors such as environmental science, food safety, pharmaceuticals, forensic science, chemical ecology, and energy production. Whether it involves detecting environmental pollutants, verifying the safety of food products, optimizing biofuel composition, or pioneering new pharmaceuticals, the demand for advanced, high-throughput analytical and data processing technologies is ubiquitous and critical across these domains.

Liquid chromatography–tandem mass spectrometry (LC-MS/MS) has been at the forefront of analytical science for the past three decades, particularly due to advances in the Orbitrap mass spectrometer and quadrupole time-of-flight (QTOF) mass analyzers. These innovations have established MS/MS as a fundamental tool for analyzing metabolites, lipids, and chemicals (Heiles 2021). Despite these technological strides, challenges in molecular characterization remain prominent. In response, our team has dedicated years to developing and integrating two pioneering technologies (Smith et al, 2006; Tautenhahn et al, 2012a; Smith et al, 2005; Tautenhahn et al, 2012a; Xue et al, 2020): XCMS and METLIN. XCMS is an informatics platform that improved metabolomic and lipidomic analysis by introducing nonlinear chromatographic alignment and data processing for enhanced statistical analysis. METLIN, on the other hand, offers a uniquely comprehensive high-resolution MS/MS database (Fig. 1), containing experimental data on over 935,000 molecular standards across more than 350 chemical classes, analyzed systematically under multiple ionization modes and collision energies (Xue et al, 2020). This database’s scale and breadth of reference data surpass any existing high-resolution resources by over an order of magnitude (a factor of 19). The integration of XCMS’s advanced data processing capabilities with METLIN’s extensive molecular standards has culminated in a unified platform that not only elevates the scope of metabolomic, lipidomic, and chemical analysis but also supports a wide array of fields that depend on precise small molecule characterization.

**Figure 1:**
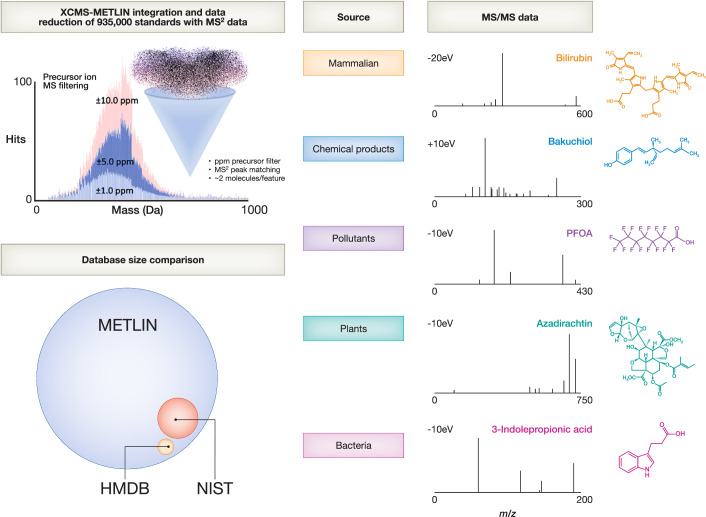
XCMS-METLIN integration and data reduction derived from precursor ion filter and MS^2^ matching. (Top left) Precursor ion filtering of METLIN’s 935,000 standards reduces the number of potential hits by 4 orders of magnitude, for example, at 10 parts per million (ppm) filtering, the number of hits is well below 100, at 5 ppm, the hits are typically below 50, and at 1 ppm the number of hits are below 20. The application of METLIN MS/MS peak matching typically drops the potential hits down to the single digits. (Lower left) The spheres represent other molecular standards-based databases, METLIN’s MS/MS database (blue), NIST (red), and HMDB (orange). XCMS-METLIN can be applied to any field of study involving small molecules, representative examples with MS/MS data include bilirubin (plasma metabolite), bakuchiol (cosmetic plant-derived metabolite), perfluorooctanoic acid (PFOA) a member of the “forever chemicals” per- and polyfluoroalkyl substance (PFAS) family of chemicals, azadirachtin (neem oil active metabolite), and 3-indolepropionic acid (IPA, microbiome-derived plasma metabolite).

The integration of XCMS and METLIN faced several significant challenges, primarily related to data processing and accurate molecular identification. A critical challenge was the harmonization of high-resolution mass spectrometry data with the extensive METLIN database. XCMS-METLIN (version 1.0.0r00) is accessible at https://xcms-metlin.scripps.edu. XCMS-METLIN integrates the XCMS data processing with the 935K METLIN database for metabolite identification. The online platform is developed using R for XCMS functionalities, with additional integration code written in Python, PHP, JavaScript, and HTML. Users can perform data analysis such as single, pairwise, and multigroup directly on the platform. The workflow involves uploading raw or converted datasets, configuring the XCMS parameters, and running the cosine scoring (Tautenhahn et al, 2012b). Once the scoring is complete, users can view the matching features within XCMS-METLIN. The user interface is designed with drag-and-drop dataset functionality, adjustable scoring settings, and straightforward visualization and filtering.

The XCMS platform’s data processing, including nonlinear chromatographic alignment and statistical analysis, were key in managing this data harmonization. Alignment and statistical analysis provided a reliable foundation for subsequent molecular identification. Furthermore, the high-throughput nature of LC-MS/MS datasets required an efficient and scalable approach to handle METLIN’s data. A key component in the integration with METLIN was the refinement of molecular identifications within a defined precursor mass window (Fig. 1 box), taking advantage of Orbitrap and QTOF high *m/z* accuracy, allowed us to significantly narrow the search scope, and enhance the speed and accuracy of the identification process. For example, as the instrumentation’s precision allows for a typical error window from 1 to 10 parts per million (ppm), serving as the preliminary filter (Fig. 1) and significantly narrowing the search scope within METLIN. By applying a ±10-ppm error window reduces METLIN’s database of 935,000 molecules to, on average, 74 intact precursor ion hits. To illustrate, when linoleic acid—a fatty acid found in a plasma extract—was filtered based on its precursor *m/z* within a ±10-ppm window, it resulted in just 22 candidate molecules. This precursor filtration markedly decreases the volume of comparisons needed in the subsequent MS/MS analysis stage. When combined with cosine scoring, the MS/MS comparison typically isolates one or two high-quality hits, further streamlining the identification process.

The integration of XCMS and METLIN has far-reaching implications across diverse scientific disciplines. In biological research, this platform offers enhanced molecular coverage and detail, leading to more profound insights into metabolic pathways and their regulation. This advancement can provide mechanistic information essential for fundamental biochemistry, drug discovery, and microbial pharmacology (e.g., microbial metabolites as illustrated in Fig. 1). In environmental science, such as the identification of PFAS (Fig. 1), the platform accurately identifies pollutants and their metabolites. In the realms of food safety and cosmetics, it facilitates the identification of a wide array of contaminants, adulterants, as well as beneficial additives (e.g., bakuchiol cosmetic additive as shown in Fig. 1). For lipid research, METLIN’s exclusive use of authenticated standards (Baker et al, 2024) makes its data uniquely valuable, enabling reliable matching against genuine standards and cross-validation with computationally generated databases. The extensive nature of METLIN is underscored by the fact that none of the representative molecules depicted in Fig. 1 are available in the NIST MS/MS database.

Both XCMS and METLIN each have an extensive history of being applied to data analysis and molecular identification, respectively. However, the integration of XCMS with the now expansive METLIN significantly enhances the ability to identify metabolites, lipids, and chemical entities across a wide spectrum of biological, pharmaceutical, and environmental matrices. This union is an important step in the progression towards digitizing chemical biology (Hudson et al, 2023; Gauthier et al, 2019), facilitating more efficient and accurate analysis.
